# Bis[1,2-bis­(dimethyl­phosphino)ethane-κ^2^ *P*,*P*′]rhodium(I) dichlorido[(1,2,5,6-η)-1,5-cyclo­octa­diene]rhodium(I)

**DOI:** 10.1107/S1600536808032509

**Published:** 2008-10-15

**Authors:** Michael Block, Santiago Gómez-Ruiz, Dirk Steinborn

**Affiliations:** a Institut für Chemie, Martin-Luther-Universität Halle-Wittenberg, Kurt-Mothes-Strasse 2, D-06120 Halle, Germany; b Departamento de Química Inorgánica y Analítica, ESCET, Universidad Rey Juan Carlos, 28933 Móstoles, Madrid, Spain

## Abstract

In the title complex, [Rh(C_6_H_16_P_2_)_2_][RhCl_2_(C_8_H_12_)], the asymmetric unit contains two [Rh(dmpe)_2_] [dmpe = 1,2-bis­(dimethyl­phosphino)ethane] half-cations, lying on inversion centers, and an [RhCl_2_(cod)]^−^ (cod = 1,5-cyclo­octa­diene) anion, wherein Rh is coordinated by two chloride ligands and two olefinic π-bonds of the cyclo­octa­diene ligand. The Rh atoms in the cations and anion exhibit square-planar coordination and are separated without any unusual inter­actions.

## Related literature

For related literature, see: Fairlie & Bosnich (1987 ▶); Wang *et al.* (2000 ▶); Cao *et al.* (2000 ▶). For a description of the Cambridge Structural Databsae, see: Allen (2002 ▶).
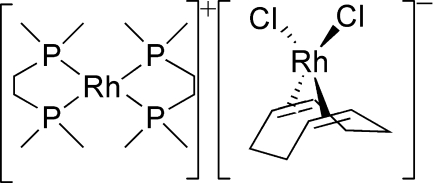


## Experimental

### 

#### Crystal data

[Rh(C_6_H_16_P_2_)_2_][RhCl_2_(C_8_H_12_)]
*M* *_r_* = 685.15
Triclinic, 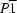

*a* = 10.4972 (2) Å
*b* = 11.2873 (4) Å
*c* = 13.0884 (4) Å
α = 71.657 (3)°
β = 80.388 (2)°
γ = 68.346 (3)°
*V* = 1365.83 (8) Å^3^
*Z* = 2
Mo *K*α radiation
μ = 1.65 mm^−1^
*T* = 130 (2) K
0.4 × 0.2 × 0.2 mm

#### Data collection

Oxford Diffraction CCD Xcalibur S diffractometer
Absorption correction: multi-scan (*CrysAlis RED*; Oxford Diffraction, 2008 ▶) *T*_min_ = 0.648, *T*_max_ = 0.720
40667 measured reflections
8309 independent reflections
6991 reflections with *I* > 2σ(*I*)
*R*_int_ = 0.027

#### Refinement

*R*[*F* ^2^ > 2σ(*F* ^2^)] = 0.022
*wR*(*F* ^2^) = 0.052
*S* = 1.01
8309 reflections
264 parameters
H-atom parameters constrained
Δρ_max_ = 1.06 e Å^−3^
Δρ_min_ = −0.74 e Å^−3^

### 

Data collection: *CrysAlis CCD* (Oxford Diffraction, 2008 ▶); cell refinement: *CrysAlis RED* (Oxford Diffraction, 2008 ▶); data reduction: *CrysAlis RED*; program(s) used to solve structure: *SHELXS97* (Sheldrick, 2008 ▶); program(s) used to refine structure: *SHELXL97* (Sheldrick, 2008 ▶); molecular graphics: *ORTEP-3* (Farrugia, 1997 ▶); software used to prepare material for publication: *SHELXL97*.

## Supplementary Material

Crystal structure: contains datablocks I, global. DOI: 10.1107/S1600536808032509/pv2102sup1.cif

Structure factors: contains datablocks I. DOI: 10.1107/S1600536808032509/pv2102Isup2.hkl

Additional supplementary materials:  crystallographic information; 3D view; checkCIF report

## Figures and Tables

**Fig. 1.**
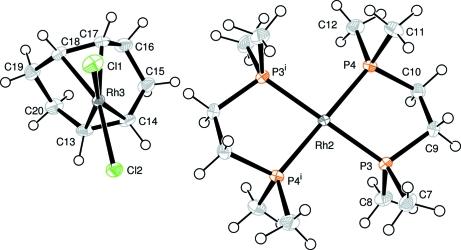
Molecular structure of [Rh(dmpe)2][RhCl2(cod)] complex. Displacement ellipsoids are plotted at the 50% probability level and H atoms are shown as small spheres of arbitrary radii. [Symmetry code: (i) -x, -y, -z]

## supplementary crystallographic information

### Comment

In the asymmetric unit of the title complex, [Rh(dmpe)_2_][RhCl_2_(cod)], one anion, [RhCl_2_(cod)]^-^, and two halves of the cation, [Rh(dmpe)_2_]^+^, were found. Each of the two [Rh(dmpe)_2_]^+^ cations exhibited crystallogaphically imposed inversion symmetry with the rhodium atoms lying on the inverison centers. The anions and the cations are separated by normal van der Waals distances without unusual interactions (shortest distance between non-hydrogen atoms: C9···Cl2^ii^ = 3.487 (2) Å, ((ii) -*x* + 1, -*y* + 1, -*z* + 1).

As expected for Rh(I) *d^8^*-complexes both the cations and the anions of the title complex exhibit a square-planar coordination of Rh. The primary coordination sphere of the [Rh(dmpe)_2_]^+^ cations is built up by four P atoms. The Rh—P bond lengths range from 2.6762 (4) (Rh1—P2) to 2.2819 (4) Å (Rh2—P4). Thus, they are relatively short compared to those in other fourfold P-coordinated Rh(I) complexes (median: 2.314 Å, lower/upper quartile: 2.289/2.342 Å, 68 observations taken from CSD version 1.10; Allen, 2002). Inspection of the torsion angles of the five-membered rings Rh1—P1—C3—C4—P2 and Rh2—P3—C9—C10—P4 exhibited conformations close to half chairs twisted on C3/C4 and C9/C10, respectively.

In the anion of the title complex, [RhCl_2_(cod)]^-^, the rhodium atom is fourfold coordinated by two chloro ligands and two olefinic π-bonds (C13/C14 and C17/C18) of the cyclooctadiene ligand. Compared to other 1,5-cyclooctadiene-coordinated Rh(I) complexes the two double bonds in the anion are slightly elongated (C13—C14 1.398 (2) Å, C17—C18 1.4012 (2) Å) (median: 1.383 Å, lower/upper quartile: 1.369/1.396 Å, 978 observations). The Rh—Cl bonds (Rh3—Cl1 2.3732 (5) Å, Rh3—Cl2 2.3807 (4) Å) are in the expected range (median: 2.391 Å, lower/upper quartile: 2.355/2.432 Å, 1812 observations taken from CSD version 1.10; Allen, 2002).

### Experimental

Under anaerobic conditions [{Rh(*µ*-Cl)(cod)}_2_] (0.20 g, 0.41 mmol) was dissolved in toluene (10 ml) at 333–343 K and to a stirred solution of 1,2-bis(dimethylphosphino)ethane (dmpe) (0.12 g, 0.82 mmol) in toluene (12 ml) was added dropwise over a period of 45 minutes. After refluxing for 3 h an orange colored solution and a yellow crystalline precipitate was obtained. Then *n*-pentane (10 ml) was added and the reaction mixture was cooled to 195 K. The precipitate was collected by filtration, washed with *n*-pentane (6 *x* 15 ml) and recrystallized from tetrahydrofuran.

### Refinement

All H atoms were positioned geometrically and treated as riding model with C–H bond distances of 0.98, 0.99 and 1.00 Å for CH_3_, CH_2_ and CH type H-atoms with U_iso_ = 1.5 times U_eq_(methyl C) and 1.2 times U_eq_(non-methyl C).

### Crystal data

**Table:**

[Rh(C_6_H_16_P_2_)_2_][RhCl_2_(C_8_H_12_)]	*Z* = 2
*M**_r_* = 685.15	*F*(000) = 696
Triclinic, *P*1	*D*_x_ = 1.666 Mg m^−^^3^
Hall symbol: -P 1	Mo *K*α radiation, λ = 0.71073 Å
*a* = 10.4972 (2) Å	Cell parameters from 19365 reflections
*b* = 11.2873 (4) Å	θ = 2.7–32.5°
*c* = 13.0884 (4) Å	µ = 1.65 mm^−^^1^
α = 71.657 (3)°	*T* = 130 K
β = 80.388 (2)°	Plates, orange
γ = 68.346 (3)°	0.4 × 0.2 × 0.2 mm
*V* = 1365.83 (8) Å^3^	

### Data collection

**Table:**

Oxford Diffraction CCD Xcalibur S diffractometer	8309 independent reflections
graphite	6991 reflections with *I* > 2σ(*I*)
Detector resolution: 16.356 pixels mm^-1^	*R*_int_ = 0.027
ω and φ scans	θ_max_ = 30.5°, θ_min_ = 2.7°
Absorption correction: multi-scan (*CrysAlis RED*; Oxford Diffraction, 2008)	*h* = −14→14
*T*_min_ = 0.648, *T*_max_ = 0.720	*k* = −16→16
40667 measured reflections	*l* = −18→18

### Refinement

**Table:**

Refinement on *F*^2^	Primary atom site location: structure-invariant direct methods
Least-squares matrix: full	Secondary atom site location: difference Fourier map
*R*[*F*^2^ > 2σ(*F*^2^)] = 0.022	Hydrogen site location: inferred from neighbouring sites
*wR*(*F*^2^) = 0.052	H-atom parameters constrained
*S* = 1.01	*w* = 1/[σ^2^(*F*_o_^2^) + (0.0295*P*)^2^] where *P* = (*F*_o_^2^ + 2*F*_c_^2^)/3
8309 reflections	(Δ/σ)_max_ = 0.003
264 parameters	Δρ_max_ = 1.06 e Å^−^^3^
0 restraints	Δρ_min_ = −0.74 e Å^−^^3^

### Special details

**Table:**

Geometry. All e.s.d.'s (except the e.s.d. in the dihedral angle between two l.s. planes) are estimated using the full covariance matrix. The cell e.s.d.'s are taken into account individually in the estimation of e.s.d.'s in distances, angles and torsion angles; correlations between e.s.d.'s in cell parameters are only used when they are defined by crystal symmetry. An approximate (isotropic) treatment of cell e.s.d.'s is used for estimating e.s.d.'s involving l.s. planes.
Refinement. Refinement of *F*^2^ against ALL reflections. The weighted *R*-factor *wR* and goodness of fit *S* are based on *F*^2^, conventional *R*-factors *R* are based on *F*, with *F* set to zero for negative *F*^2^. The threshold expression of *F*^2^ > σ(*F*^2^) is used only for calculating *R*-factors(gt) *etc*. and is not relevant to the choice of reflections for refinement. *R*-factors based on *F*^2^ are statistically about twice as large as those based on *F*, and *R*- factors based on ALL data will be even larger.

### Fractional atomic coordinates and isotropic or equivalent isotropic displacement parameters (Å^2^)

**Table:**

	*x*	*y*	*z*	*U*_iso_*/*U*_eq_	
Rh1	0	0	0	0.01449 (4)	
Rh2	0.5	0.5	0.5	0.01465 (4)	
Rh3	0.789115 (13)	0.818563 (12)	0.730707 (9)	0.01649 (4)	
Cl2	0.64019 (5)	0.72031 (4)	0.85695 (3)	0.02505 (9)	
Cl1	0.97386 (5)	0.61380 (4)	0.76211 (4)	0.02901 (10)	
P1	0.01483 (5)	−0.21735 (4)	0.05439 (3)	0.01713 (8)	
P2	0.22397 (4)	−0.08105 (4)	−0.05722 (3)	0.01782 (9)	
P3	0.31544 (5)	0.44601 (4)	0.48695 (3)	0.01834 (9)	
P4	0.54933 (5)	0.49466 (4)	0.32464 (3)	0.01772 (9)	
C2	0.0328 (2)	−0.28727 (18)	0.19860 (13)	0.0246 (4)	
H2A	−0.053	−0.2473	0.2371	0.037*	
H2B	0.1077	−0.2689	0.2193	0.037*	
H2C	0.0535	−0.3834	0.2174	0.037*	
C12	0.6066 (2)	0.6161 (2)	0.21891 (14)	0.0298 (4)	
H12A	0.7035	0.5997	0.2264	0.045*	
H12B	0.5515	0.7054	0.2249	0.045*	
H12C	0.5958	0.6089	0.1484	0.045*	
C3	0.17446 (19)	−0.31739 (18)	−0.00272 (15)	0.0271 (4)	
H3A	0.2007	−0.4108	0.0419	0.033*	
H3B	0.1612	−0.315	−0.0766	0.033*	
C5	0.25655 (19)	−0.05513 (19)	−0.20196 (13)	0.0266 (4)	
H5A	0.2371	0.0397	−0.2374	0.04*	
H5B	0.1972	−0.0871	−0.2288	0.04*	
H5C	0.3529	−0.104	−0.2177	0.04*	
C8	0.15174 (19)	0.5794 (2)	0.48305 (16)	0.0299 (4)	
H8A	0.1616	0.6622	0.4348	0.045*	
H8B	0.1222	0.5899	0.5557	0.045*	
H8C	0.0831	0.5583	0.4565	0.045*	
C10	0.39535 (18)	0.50045 (19)	0.27240 (13)	0.0235 (4)	
H10A	0.3298	0.5924	0.2545	0.028*	
H10B	0.4201	0.4707	0.206	0.028*	
C7	0.2741 (2)	0.3057 (2)	0.58132 (15)	0.0320 (4)	
H7A	0.2321	0.3284	0.6484	0.048*	
H7B	0.3583	0.2286	0.5967	0.048*	
H7C	0.2097	0.2847	0.5496	0.048*	
C11	0.6754 (2)	0.34147 (19)	0.30462 (15)	0.0287 (4)	
H11A	0.6793	0.3424	0.229	0.043*	
H11B	0.6492	0.2661	0.3511	0.043*	
H11C	0.7657	0.3332	0.3228	0.043*	
C1	−0.1096 (2)	−0.27999 (19)	0.02981 (16)	0.0315 (4)	
H1A	−0.0742	−0.3772	0.0521	0.047*	
H1B	−0.1262	−0.2484	−0.0472	0.047*	
H1C	−0.1958	−0.2483	0.0713	0.047*	
C9	0.32987 (19)	0.41001 (19)	0.35813 (13)	0.0237 (4)	
H9A	0.3868	0.3161	0.3647	0.028*	
H9B	0.2377	0.4245	0.3374	0.028*	
C13	0.65456 (18)	1.01014 (17)	0.73195 (14)	0.0242 (4)	
H13	0.5873	1.0124	0.7958	0.029*	
C14	0.62098 (18)	0.96957 (18)	0.65258 (14)	0.0243 (4)	
H14	0.5339	0.9486	0.6703	0.029*	
C15	0.6539 (2)	1.0163 (2)	0.53370 (15)	0.0316 (4)	
H15A	0.5932	1.109	0.5054	0.038*	
H15B	0.635	0.961	0.4966	0.038*	
C19	0.8837 (2)	1.04352 (18)	0.71184 (16)	0.0273 (4)	
H19A	0.9289	1.1046	0.662	0.033*	
H19B	0.9108	1.0257	0.7856	0.033*	
C18	0.93356 (18)	0.91525 (17)	0.68116 (14)	0.0214 (3)	
H18	1.0286	0.8572	0.7029	0.026*	
C6	0.3591 (2)	−0.0369 (2)	−0.02436 (17)	0.0354 (5)	
H6A	0.4487	−0.0996	−0.0395	0.053*	
H6B	0.3484	−0.0403	0.0522	0.053*	
H6C	0.3529	0.053	−0.0681	0.053*	
C20	0.72756 (19)	1.11091 (18)	0.70816 (16)	0.0298 (4)	
H20A	0.6943	1.1626	0.7618	0.036*	
H20B	0.705	1.1734	0.6359	0.036*	
C17	0.89609 (18)	0.89728 (18)	0.59071 (13)	0.0222 (3)	
H17	0.969	0.8289	0.56	0.027*	
C4	0.28710 (19)	−0.26187 (18)	−0.00563 (14)	0.0268 (4)	
H4A	0.3663	−0.2996	−0.0524	0.032*	
H4B	0.3186	−0.2876	0.0679	0.032*	
C16	0.80332 (19)	1.0096 (2)	0.50800 (14)	0.0303 (4)	
H16A	0.8334	0.996	0.4355	0.036*	
H16B	0.811	1.0947	0.5071	0.036*	

### Atomic displacement parameters (Å^2^)

**Table:**

	*U*^11^	*U*^22^	*U*^33^	*U*^12^	*U*^13^	*U*^23^
Rh1	0.01354 (9)	0.01490 (8)	0.01477 (7)	−0.00652 (7)	0.00139 (6)	−0.00282 (6)
Rh2	0.01290 (9)	0.01807 (8)	0.01478 (7)	−0.00699 (7)	0.00143 (6)	−0.00598 (6)
Rh3	0.01552 (7)	0.01655 (6)	0.01915 (6)	−0.00667 (5)	0.00059 (5)	−0.00661 (5)
Cl2	0.0255 (2)	0.0272 (2)	0.02541 (19)	−0.01429 (18)	0.00422 (16)	−0.00774 (16)
Cl1	0.0233 (2)	0.01754 (19)	0.0422 (2)	−0.00484 (17)	−0.00048 (19)	−0.00613 (17)
P1	0.0186 (2)	0.01635 (19)	0.01718 (17)	−0.00832 (17)	0.00025 (15)	−0.00343 (15)
P2	0.0150 (2)	0.0210 (2)	0.01775 (17)	−0.00742 (17)	0.00198 (15)	−0.00553 (15)
P3	0.0162 (2)	0.0212 (2)	0.02015 (18)	−0.00926 (18)	0.00094 (16)	−0.00652 (16)
P4	0.0175 (2)	0.0198 (2)	0.01589 (17)	−0.00665 (17)	0.00181 (15)	−0.00596 (15)
C2	0.0313 (11)	0.0228 (8)	0.0199 (7)	−0.0120 (8)	−0.0015 (7)	−0.0026 (6)
C12	0.0336 (11)	0.0341 (10)	0.0217 (8)	−0.0182 (9)	0.0029 (7)	−0.0018 (7)
C3	0.0291 (11)	0.0182 (8)	0.0290 (8)	−0.0050 (7)	0.0062 (7)	−0.0071 (7)
C5	0.0250 (10)	0.0310 (10)	0.0191 (7)	−0.0071 (8)	0.0036 (7)	−0.0061 (7)
C8	0.0185 (10)	0.0346 (10)	0.0357 (10)	−0.0074 (8)	0.0003 (8)	−0.0113 (8)
C10	0.0229 (10)	0.0284 (9)	0.0204 (7)	−0.0084 (8)	−0.0037 (7)	−0.0076 (7)
C7	0.0364 (12)	0.0341 (10)	0.0317 (9)	−0.0238 (9)	0.0007 (8)	−0.0046 (8)
C11	0.0276 (11)	0.0274 (9)	0.0276 (8)	−0.0040 (8)	0.0035 (7)	−0.0119 (7)
C1	0.0352 (12)	0.0270 (9)	0.0384 (10)	−0.0170 (9)	−0.0101 (9)	−0.0054 (8)
C9	0.0228 (10)	0.0296 (9)	0.0251 (8)	−0.0124 (8)	−0.0025 (7)	−0.0115 (7)
C13	0.0195 (9)	0.0194 (8)	0.0308 (8)	−0.0046 (7)	0.0067 (7)	−0.0094 (7)
C14	0.0144 (9)	0.0271 (9)	0.0273 (8)	−0.0067 (7)	0.0008 (7)	−0.0035 (7)
C15	0.0220 (10)	0.0426 (12)	0.0264 (8)	−0.0129 (9)	−0.0045 (7)	−0.0003 (8)
C19	0.0282 (11)	0.0218 (9)	0.0360 (9)	−0.0126 (8)	−0.0015 (8)	−0.0087 (7)
C18	0.0149 (9)	0.0188 (8)	0.0301 (8)	−0.0067 (7)	0.0004 (7)	−0.0061 (7)
C6	0.0204 (10)	0.0505 (13)	0.0474 (12)	−0.0162 (10)	0.0028 (9)	−0.0270 (10)
C20	0.0277 (11)	0.0203 (8)	0.0416 (10)	−0.0077 (8)	0.0041 (8)	−0.0125 (8)
C17	0.0167 (9)	0.0251 (8)	0.0232 (7)	−0.0085 (7)	0.0054 (6)	−0.0060 (7)
C4	0.0211 (10)	0.0230 (9)	0.0262 (8)	−0.0026 (7)	0.0035 (7)	−0.0015 (7)
C16	0.0235 (10)	0.0387 (11)	0.0234 (8)	−0.0114 (9)	0.0005 (7)	−0.0011 (8)

### Geometric parameters (Å, °)

**Table:**

Rh1—P2	2.2762 (4)	C8—H8C	0.98
Rh1—P2^i^	2.2762 (4)	C10—C9	1.521 (2)
Rh1—P1	2.2815 (4)	C10—H10A	0.99
Rh1—P1^i^	2.2815 (4)	C10—H10B	0.99
Rh2—P3^ii^	2.2807 (4)	C7—H7A	0.98
Rh2—P3	2.2807 (4)	C7—H7B	0.98
Rh2—P4	2.2819 (4)	C7—H7C	0.98
Rh2—P4^ii^	2.2819 (4)	C11—H11A	0.98
Rh3—C14	2.0860 (18)	C11—H11B	0.98
Rh3—C18	2.0876 (17)	C11—H11C	0.98
Rh3—C13	2.1051 (17)	C1—H1A	0.98
Rh3—C17	2.1053 (16)	C1—H1B	0.98
Rh3—Cl1	2.3732 (5)	C1—H1C	0.98
Rh3—Cl2	2.3807 (4)	C9—H9A	0.99
P1—C1	1.8096 (18)	C9—H9B	0.99
P1—C2	1.8145 (17)	C13—C14	1.398 (2)
P1—C3	1.8326 (18)	C13—C20	1.523 (2)
P2—C5	1.8167 (16)	C13—H13	1
P2—C6	1.8177 (19)	C14—C15	1.502 (2)
P2—C4	1.8288 (18)	C14—H14	1
P3—C8	1.8150 (19)	C15—C16	1.526 (3)
P3—C7	1.8214 (19)	C15—H15A	0.99
P3—C9	1.8251 (17)	C15—H15B	0.99
P4—C11	1.8138 (18)	C19—C18	1.506 (2)
P4—C12	1.8146 (18)	C19—C20	1.534 (3)
P4—C10	1.8295 (18)	C19—H19A	0.99
C2—H2A	0.98	C19—H19B	0.99
C2—H2B	0.98	C18—C17	1.401 (2)
C2—H2C	0.98	C18—H18	1
C12—H12A	0.98	C6—H6A	0.98
C12—H12B	0.98	C6—H6B	0.98
C12—H12C	0.98	C6—H6C	0.98
C3—C4	1.520 (3)	C20—H20A	0.99
C3—H3A	0.99	C20—H20B	0.99
C3—H3B	0.99	C17—C16	1.518 (2)
C5—H5A	0.98	C17—H17	1
C5—H5B	0.98	C4—H4A	0.99
C5—H5C	0.98	C4—H4B	0.99
C8—H8A	0.98	C16—H16A	0.99
C8—H8B	0.98	C16—H16B	0.99
			
P2—Rh1—P2^i^	180.00 (2)	H10A—C10—H10B	108.4
P2—Rh1—P1	84.146 (16)	P3—C7—H7A	109.5
P2^i^—Rh1—P1	95.854 (16)	P3—C7—H7B	109.5
P2—Rh1—P1^i^	95.854 (16)	H7A—C7—H7B	109.5
P2^i^—Rh1—P1^i^	84.146 (16)	P3—C7—H7C	109.5
P1—Rh1—P1^i^	180	H7A—C7—H7C	109.5
P3^ii^—Rh2—P3	180.000 (7)	H7B—C7—H7C	109.5
P3^ii^—Rh2—P4	95.774 (15)	P4—C11—H11A	109.5
P3—Rh2—P4	84.226 (15)	P4—C11—H11B	109.5
P3^ii^—Rh2—P4^ii^	84.226 (15)	H11A—C11—H11B	109.5
P3—Rh2—P4^ii^	95.774 (15)	P4—C11—H11C	109.5
P4—Rh2—P4^ii^	180	H11A—C11—H11C	109.5
C14—Rh3—C18	98.94 (7)	H11B—C11—H11C	109.5
C14—Rh3—C13	38.96 (7)	P1—C1—H1A	109.5
C18—Rh3—C13	82.54 (7)	P1—C1—H1B	109.5
C14—Rh3—C17	81.97 (7)	H1A—C1—H1B	109.5
C18—Rh3—C17	39.04 (7)	P1—C1—H1C	109.5
C13—Rh3—C17	90.89 (7)	H1A—C1—H1C	109.5
C14—Rh3—Cl1	156.67 (5)	H1B—C1—H1C	109.5
C18—Rh3—Cl1	88.31 (5)	C10—C9—P3	109.25 (11)
C13—Rh3—Cl1	163.98 (5)	C10—C9—H9A	109.8
C17—Rh3—Cl1	90.22 (5)	P3—C9—H9A	109.8
C14—Rh3—Cl2	90.68 (5)	C10—C9—H9B	109.8
C18—Rh3—Cl2	155.46 (5)	P3—C9—H9B	109.8
C13—Rh3—Cl2	91.23 (5)	H9A—C9—H9B	108.3
C17—Rh3—Cl2	165.43 (5)	C14—C13—C20	123.88 (16)
Cl1—Rh3—Cl2	91.696 (16)	C14—C13—Rh3	69.78 (10)
C1—P1—C2	102.79 (9)	C20—C13—Rh3	113.15 (12)
C1—P1—C3	101.99 (9)	C14—C13—H13	114.1
C2—P1—C3	103.04 (9)	C20—C13—H13	114.1
C1—P1—Rh1	125.04 (7)	Rh3—C13—H13	114.1
C2—P1—Rh1	111.70 (6)	C13—C14—C15	125.14 (16)
C3—P1—Rh1	109.87 (6)	C13—C14—Rh3	71.26 (10)
C5—P2—C6	101.34 (9)	C15—C14—Rh3	111.50 (12)
C5—P2—C4	102.65 (9)	C13—C14—H14	113.8
C6—P2—C4	102.09 (10)	C15—C14—H14	113.8
C5—P2—Rh1	115.55 (6)	Rh3—C14—H14	113.8
C6—P2—Rh1	123.10 (7)	C14—C15—C16	112.46 (15)
C4—P2—Rh1	109.57 (6)	C14—C15—H15A	109.1
C8—P3—C7	101.85 (10)	C16—C15—H15A	109.1
C8—P3—C9	103.17 (9)	C14—C15—H15B	109.1
C7—P3—C9	102.03 (9)	C16—C15—H15B	109.1
C8—P3—Rh2	114.62 (7)	H15A—C15—H15B	107.8
C7—P3—Rh2	123.37 (7)	C18—C19—C20	112.53 (15)
C9—P3—Rh2	109.43 (6)	C18—C19—H19A	109.1
C11—P4—C12	100.84 (9)	C20—C19—H19A	109.1
C11—P4—C10	102.58 (9)	C18—C19—H19B	109.1
C12—P4—C10	103.19 (9)	C20—C19—H19B	109.1
C11—P4—Rh2	115.09 (6)	H19A—C19—H19B	107.8
C12—P4—Rh2	124.40 (6)	C17—C18—C19	125.41 (17)
C10—P4—Rh2	108.19 (5)	C17—C18—Rh3	71.16 (9)
P1—C2—H2A	109.5	C19—C18—Rh3	111.01 (12)
P1—C2—H2B	109.5	C17—C18—H18	113.8
H2A—C2—H2B	109.5	C19—C18—H18	113.8
P1—C2—H2C	109.5	Rh3—C18—H18	113.8
H2A—C2—H2C	109.5	P2—C6—H6A	109.5
H2B—C2—H2C	109.5	P2—C6—H6B	109.5
P4—C12—H12A	109.5	H6A—C6—H6B	109.5
P4—C12—H12B	109.5	P2—C6—H6C	109.5
H12A—C12—H12B	109.5	H6A—C6—H6C	109.5
P4—C12—H12C	109.5	H6B—C6—H6C	109.5
H12A—C12—H12C	109.5	C13—C20—C19	111.66 (15)
H12B—C12—H12C	109.5	C13—C20—H20A	109.3
C4—C3—P1	108.96 (12)	C19—C20—H20A	109.3
C4—C3—H3A	109.9	C13—C20—H20B	109.3
P1—C3—H3A	109.9	C19—C20—H20B	109.3
C4—C3—H3B	109.9	H20A—C20—H20B	107.9
P1—C3—H3B	109.9	C18—C17—C16	122.95 (16)
H3A—C3—H3B	108.3	C18—C17—Rh3	69.79 (9)
P2—C5—H5A	109.5	C16—C17—Rh3	113.68 (11)
P2—C5—H5B	109.5	C18—C17—H17	114.2
H5A—C5—H5B	109.5	C16—C17—H17	114.2
P2—C5—H5C	109.5	Rh3—C17—H17	114.2
H5A—C5—H5C	109.5	C3—C4—P2	110.47 (13)
H5B—C5—H5C	109.5	C3—C4—H4A	109.6
P3—C8—H8A	109.5	P2—C4—H4A	109.6
P3—C8—H8B	109.5	C3—C4—H4B	109.6
H8A—C8—H8B	109.5	P2—C4—H4B	109.6
P3—C8—H8C	109.5	H4A—C4—H4B	108.1
H8A—C8—H8C	109.5	C17—C16—C15	111.14 (15)
H8B—C8—H8C	109.5	C17—C16—H16A	109.4
C9—C10—P4	108.60 (12)	C15—C16—H16A	109.4
C9—C10—H10A	110	C17—C16—H16B	109.4
P4—C10—H10A	110	C15—C16—H16B	109.4
C9—C10—H10B	110	H16A—C16—H16B	108
P4—C10—H10B	110		

Symmetry codes: (i) −*x*, −*y*, −*z*; (ii) −*x*+1, −*y*+1, −*z*+1.
